# *CmRCC1* Gene From Pumpkin Confers Cold Tolerance in Tobacco by Modulating Root Architecture and Photosynthetic Activity

**DOI:** 10.3389/fpls.2021.765302

**Published:** 2021-12-03

**Authors:** Mengmeng Wang, Shu Zhou, Junyang Lu, Anqi Xu, Yuan Huang, Zhilong Bie, Fei Cheng

**Affiliations:** Key Laboratory of Horticultural Plant Biology, Ministry of Education/College of Horticulture and Forestry Sciences, Huazhong Agricultural University, Wuhan, China

**Keywords:** *CmRCC1*, cold stress, root architecture, photosynthesis, pumpkin

## Abstract

Low-temperature stress is the main limiting factor of cucurbit crop cultivation as it affects crop yield and quality. The identification of genes involved in cold tolerance is a crucial aspect of pumpkin rootstock breeding. Here, we examined the function of a pumpkin Regulator of Chromosome Condensation 1 (*CmRCC1*) gene in the root development and cold stress responses of tobacco (*Nicotiana benthamiana*). *CmRCC1* expression was differentially induced in pumpkin root, stem, and leaf under cold stress. Transient transformation showed that CmRCC1 is located in the nucleus. *CmRCC1* overexpression in tobacco increased the gravitropic set-point angle in lateral roots, as well as root diameter and volume. The expression of auxin polar transport factors, *PIN1* and *PIN3*, decreased and increased in *CmRCC1*-overexpressed plants, respectively. Yeast two-hybrid verification and luciferase complementation imaging assay showed that CmRCC1 interacts with CmLAZY1. Furthermore, the decreases in maximum quantum yield of PS II, the effective quantum yield of PS II, and electron transfer rate and the increases in quantum yield of nonregulated energy dissipation and malondialdehyde content were compromised in transgenic plants compared with wild-type plants under cold stress. The results suggest that *CmRCC1* plays an important role in the regulation of root architecture and positively modulates cold tolerance.

## Introduction

The Regulator of Chromosome Condensation 1 (RCC1) superfamily of proteins is characterized by 350–500 residue domain, known as the RCC1-like domain (RLD), which was first reported in human RCC1 in 1987 (Ohtsubo et al., 1987). RCC1 consists of seven homologous repeats of 51–68 amino acid residues. It combines with chromatin and a nuclear Ras-like G protein, Ran, to establish a RanGTP concentration gradient, which affects the formation and function of the nuclear envelope, spindle formation, nuclear transport, and the cell cycle during tumorigenesis (Ren et al., 2020). Since the initial identification of RCC1, a number of proteins that contain one or more RLDs have been discovered. In human cells, these RCC1 superfamily proteins can be subdivided into five subgroups based on structural criteria (Hadjebi et al., 2008).

Recent studies have been reported the functions of RCC1 superfamily proteins in plants. *Arabidopsis thaliana* contains 24 RCC1 family proteins, among which UV RESISTANCE LOCUS 8 (UVR8), a UV-B photoreceptor, has been studied the most (Rizzini et al., 2011; Christie et al., 2012; Wu et al., 2012; Jenkins, 2014). UV-B absorption induces the instant monomerization of UV-B RESISTANCE 8 (UVR8) and interaction with CONSTITUTIVELY PHOTOMORPHOGENIC 1, the central regulator of light signaling, to secure plant acclimation and promote survival in sunlight (Rizzini et al., 2011). RCC1/UVR8/GEF-like 3 (RUG3), another RCC1 family protein, interacts with ataxia telangiectasia-mutated protein in the mitochondria of *Arabidopsis* to synergistically regulate *nad2* mRNA splicing and complex I biogenesis (Kühn et al., 2011). As an upstream regulatory element of reactive oxygen species (ROS) homeostasis, RUG3-mediated mitochondrial retrograde signaling plays an important role in DNA damage repair and mitochondrial function restoration in the root apical meristem (Su et al., 2017). The *Tolerant to Chilling and Freezing 1* (*TCF1)* gene in *Arabidopsis* encodes a protein containing six predicted tandem RCC1 repeats that show a similarity to yeast and human RCC1 (Ohtsubo et al., 1989; Renault et al., 1998). *TCF1* regulates cold acclimation and freezing tolerance by modulating *Blue-Copper-Binding gene* (*BCB*) to adjust lignin accumulation and consequently cell wall remodeling (Ji et al., 2015). *Sensitive to ABA 1* (*SAB1*) encodes a RCC1 family protein and physically interacts with ABI5, which results in reduced ABI5 phosphorylation and protein stability, decreased ABI5 DNA-binding activity, and increased the H3K27m2 methylation of *ABI5* promoter in *Arabidopsis* (Ji et al., 2019). Four out of eight RLD proteins in *Arabidopsis* were identified as LAZY1/LAZY1-LIKE (LZY) interactors, and RLDs regulate PIN-dependent auxin transport in various developmental processes, including gravitropic set-point angle (GSA) control (Furutani et al., 2020). A newly discovered RCC1 family protein, PLASTICITY OF ROSETTE TO NITROGEN 1, confers the plasticity of rosette diameter in response to changes in nitrogen availability in *Arabidopsis* (Duarte et al., 2021). Additionally, 56 *RCC1* genes have been identified in upland cotton (*Gossypium hirsutum*), among which *Gh_A05G3028* and *Gh_D10G2310*, the homologous genes of *AtTCF1* and *AtUVR8*, were dramatically induced under salt treatment, and the silencing of these two genes exhibited a salt-sensitive phenotype (Liu et al., 2019).

As the most important environmental stress, low temperature can limit the growth of plants and affect the distribution and yield of crops (Stitt and Hurry, 2002; Zhang et al., 2004). Low-temperature stress negatively affects plant growth morphology, physiology, and biochemistry by limiting cell survival, cell division, photosynthetic efficiency, and water transport (Beck et al., 2007; Sanghera et al., 2011). In recent years, extreme weather occurs frequently around the world and further increases the risk of low-temperature damage to plants, which remarkably reduces the economic benefits of agricultural production. Solving the adaptation problem of plants under chilling injury has always been a hot topic worldwide (Rigby and Porporato, 2008; Augspurger, 2013; Hatfield and Prueger, 2015). Therefore, studying the response mechanism of plants to chilling injury and discovering the functional genes of plants for cold resistance are of great importance to cope with global climate anomalies.

Pumpkin (*Cucurbita maxima*) is a typical warm-loving vegetable. It is often used as the rootstock in grafting many kinds of cucurbit crops because of its developed root system and strong resistance to soil-borne pathogens and abiotic stresses. Pumpkin rootstocks can reduce water loss by limiting the transpiration of grafted seedlings, promote the absorption and transportation of water and nutrients in grafted seedlings, and regulate the osmotic pressure in cells to alleviate the damage of plants under low-temperature stress (Schwarz et al., 2010). However, the possible molecular regulatory mechanisms underlying pumpkin response to cold stress are not yet illustrated. In this study, the Regulator of Chromosome Condensation 1 (*CmRCC1*) gene was characterized from a cold-tolerant pumpkin rootstock. The expression patterns of *CmRCC1* in response to cold treatment were analyzed through quantitative real-time polymerase chain reaction (qRT-PCR). *CmRCC1* was overexpressed in transgenic tobacco (*Nicotiana benthamiana*) plants to evaluate its function in root development and cold stress tolerance. Root morphology assays revealed that *CmRCC1* overexpression altered the root architecture under normal growth conditions. Moreover, *CmRCC1*-overexpressed (*OxCmRCC1*) plants showed good performance under cold stress. Generally, our results suggest that *CmRCC1* plays important roles in plant cold response and can be a candidate gene to improve the cold tolerance of crops in the future.

## Materials and Methods

### Plant Materials and Cold Treatment of Pumpkin Seedlings

“Qingyan No. 1,” a pumpkin rootstock with low temperature tolerance, was used as the experimental material in this study. The pumpkin seeds were soaked with 1‰ KMnO_4_ for 15min to conduct surface disinfection. Afterward, the seeds were soaked in warm water at 55°C, cooled naturally, soaked for 12h, and placed in a growth chamber at 30°C for germination. Then, the seeds were sown in 10cm×10cm pots with peat–vermiculite–perlite medium (2:1:1). The growth conditions were as follows: photoperiod, 12/12h; day/night temperature, 28/18°C; light intensity, 16,000 Lx; and air humidity, 70–85%. Pumpkin seedlings at three-leaf stage were exposed to 4°C in a growth chamber (Ningbo Saifu DGX-260, China) for cold stress. The root, stem, and third true leaf of each plant were sampled at 0, 3, 6, 12, and 24h after low-temperature treatment. The samples were frozen at −80°C in liquid nitrogen before qRT-PCR analysis.

### Subcellular Localization of CmRCC1

The full-length coding sequence (CDS) of *CmRCC1* was amplified by PCR using 2× High-Fidelity Master Mix (Tsingke, Inc., Beijing, China), and the fragments were inserted into the *Bgl* II site of the pCAMBIA1305.4-N-GFP vector by using ClonExpress II One Step Cloning Kits (Vazyme, Piscataway, NJ, United States) to generate *35S*::GFP-CmRCC1 fusion protein under the control of the Cauliflower mosaic virus (CaMV) 35S promoter. The construct and negative control (pCAMBIA1305.4-N-GFP) were transformed into *Agrobacterium tumefaciens* strain GV3101 and infiltrated into tobacco leaves according to previously described method (Sheludko et al., 2007). Leica SP8 confocal microscope was used to detect the GFP fluorescence signal with 4,6-diamidino-2-phenylindole (DAPI) as the nucleus marker.

### Total RNA Extraction and Reverse Transcription

Total RNA was isolated using TransZol reagent (TransGen Biotech Inc., Beijing, China) in accordance with the manufacturer’s protocol. The extracted total RNA was dissolved in diethylpyrocarbonate-treated water. The cDNA template for gene cloning was synthesized from 2μg of RNA using HiScript II One Step RT-PCR Kit (Vazyme, Piscataway, NJ, United States). While for qRT-PCR, the cDNA was synthesized from 1μg total RNA using HiScript II Q RT SuperMix for qPCR (+g DNA wiper; Vazyme, Piscataway, NJ, United States).

### Generation of *CmRCC1* Transgenic Tobacco Plants

The CDS of *CmRCC1* was cloned into the pHellgate8 vector to generate the *35S*::*CmRCC1* construct by ClonExpress II One Step Cloning Kits. The construct was transformed into *A. tumefaciens* strain GV3101 and then transferred into tobacco plants using the leaf disc method (Horsch et al., 1985). Transgenic tobacco seeds were screened on MS medium suspended with kanamycin (50mg/L). T_2_ homozygous lines were used for further experiments.

### Root Morphology Assays

The roots of three uniform plants from each replicate were harvested and washed with deionized water. The root morphology was scanned using Imagery Scan Screen (Epson Expression 11000XL, Regent Instruments, Canada). Root image analysis was conducted *via* the WinRHIZO 2003a software (Regent Instruments, Canada).

### Yeast Two-Hybrid Verification

The open reading frames (ORFs) of *CmRCC1* and *CmLAZY1* from “Qianyan No. 1” roots were amplified using sequence-specific primers (Supplementary Table S1) and incorporated into pGBKT7 and pGADT7 vectors (Clontech, United States), respectively, to verify the protein–protein interactions of CmRCC1 with CmLAZY1. According to the manufacturer, the recombinant plasmids, pGADT7-CmLAZY1 and pGBKT7-CmRCC1, pGADT7 and pGBKT7-CmRCC1, pGADT7-T and pGBKT7-lam (negative control), and pGADT7-T and pGBKT7-p53 (positive control), were introduced into the yeast strain, Y2H Gold. The transformants were grown on SD/−Leu/−Trp and SD/−Leu/−Trp/−Ade/-His media to evaluate the interactions.

### Luciferase Complementation Imaging Assay

As described previously, the ORF of *CmLAZY1* was cloned into pCAMBIA-nLUC to yield the fusion construct, pCAMBIA-CmLAZY1-nLUC, and the ORF of *CmRCC1* was cloned into pCAMBIA-cLUC to generate the fusion construct, pCAMBIA-CmRCC1-cLUC (Chen et al., 2008). *Agrobacterium tumefaciens* GV3101 was transformed with the empty vector and fusion constructs and incubated at 28°C for 16h. Then, the *A. tumefaciens* cells were collected and resuspended at OD_600_=0.3. The tobacco leaves were then infiltrated with *Agrobacterium* strains containing the indicated constructs at a ratio of 1:1. After 3days, the leaves were treated with luciferin, and firefly luciferase (LUC) signal was observed according to Xiong et al. (2019).

### Analysis of Chlorophyll Fluorescence

Chlorophyll fluorescence was measured by pulse amplitude-modulated fluorometry (MAXI; Heinz Walz, Effeltrich, Germany) as previously described (Cheng et al., 2016). The seedlings were adapted to the dark for at least 30min before the measurements, and the whole area of the third leaf from the bottom was used for the experiment. The intensities of actinic light and saturating light were set to 280 and 4,000μmolm^−2^s^−1^, respectively. The maximum quantum yield of PS II (*F*v/*F*m) and the effective quantum yield of PS II (*Φ*_PSII_) were measured and calculated in accordance with the following equations (van Kooten and Snel, 1990): *F*v/*F*m=(*F*m−*F*o)/*F*m and *Φ*_PSII_=(*F*’m−*F*s)/*F*’m. The quantum yield of regulated energy dissipation (*Φ*_NPQ_) and the quantum yield of nonregulated energy dissipation (*Φ*_NO_) in PS II were calculated according to the equation (Kramer et al., 2004): *Φ*_PSII_+*Φ*_NPQ_+*Φ*_NO_=1. Electron transfer rate (ETR) was measured using a rapid light-response curve.

### Determination of Lipid Peroxidation

Lipid peroxidation was determined by measuring malondialdehyde (MDA) content as described by Hodges et al. (1999). Briefly, leaf samples (0.3g) were ground in 3ml of ice-cold 25mmol/L HEPES buffer (pH 7.8) containing 0.2mmol/L EDTA and 2% (w/v) polyvinylpyrrolidone. The obtained homogenates were centrifuged at 4°C for 20min at 10,000rpm, and the resulting supernatants were used to analyze MDA content. The samples were mixed with 10% trichloroacetic acid containing 0.65% 2-thiobarbituric acid (TBA) and heated at 95°C for 25min. MDA content was corrected for non-MDA compounds by subtracting the absorbance at 532nm of a TBA-less solution that contained the plant extract.

### Gene Expression Analysis

We amplified the PCR products for qRT-PCR analysis in triplicate using 2×TransStart™ TOP Green qPCR SuperMix (TransGen Biotech Inc., Beijing, China) in 10μl qRT-PCR assays. PCR was performed using the QuantStudio 7 Flex Real-time PCR System (Applied Biosystems, Foster City, CA, United States). The cycling conditions consisted of denaturation at 95°C for 30s, followed by 40cycles of denaturation at 95°C for 5s, annealing at 58°C for 15s, and extension at 72°C for 10s. The reference genes, *CmCAC* and *NbACTIN*, were used as the internal controls (Obrero et al., 2011; Nie et al., 2020). The gene-specific primers for *CmRCC1* and the *NbPIN* gene family are listed in Supplementary Table S1. Relative gene expression was determined as previously described by Livak and Schmittgen (2001).

### Statistical Analysis

The experiment involved a completely randomized block design with four replicates. Statistical analysis was performed using the SAS statistical package. The differences between the treatment means were separated using Tukey’s test at a significance level of *p*<0.05.

## Results

### Identification and Characterization of the *CmRCC1* Gene

**Figure 1:**
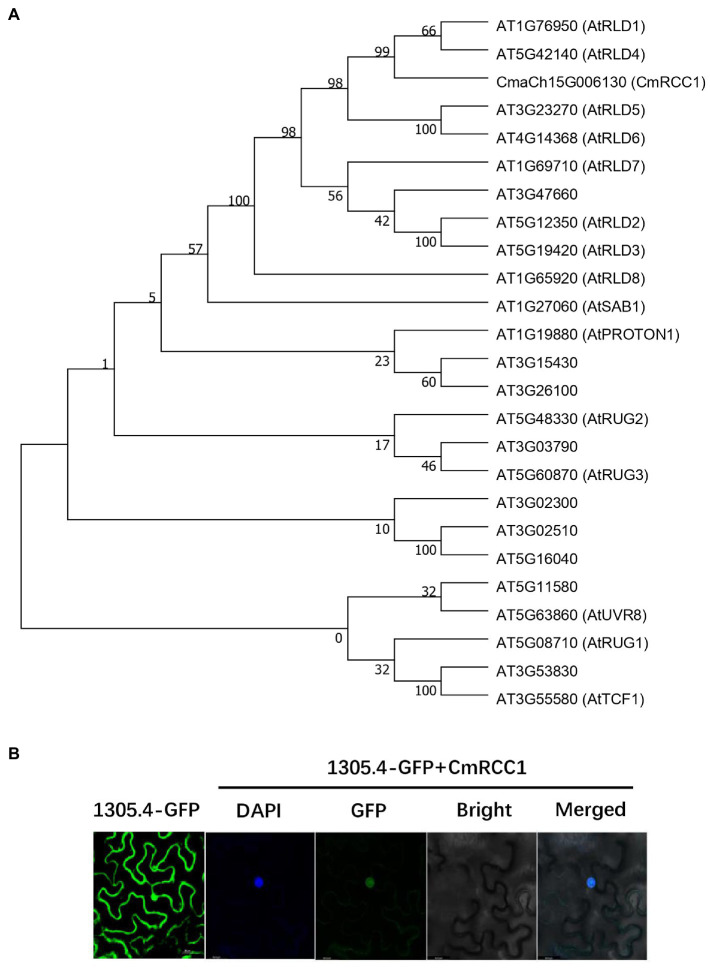
Phylogenetic analysis of RCC1 family proteins in *Arabidopsis* and subcellular localization of CmRCC1. **(A)** Phylogenetic tree of CmRCC1 with those identified RCC1 proteins from *Arabidopsis*. The phylogenetic tree was constructed using MEGA 7 with the Neighbor–Joining method. **(B)** Subcellular localization of CmRCC1 in tobacco epidermal cells. Nucleus was stained with DAPI. Co-localization between DAPI and GFP signals in *35S*::GFP-CmRCC1 fusion protein was shown in merged picture.

*CmRCC1* gene (CmaCh15G006130) was predicted to contain a 3,360bp CDS isolated from 4,143bp cDNA and encode the protein of 1,119 amino acids in the Cucurbit Genomics Database. A Pfam domain search was performed to characterize the pleckstrin homology (PH_12), RCC1 repeats, FYVE zinc finger, BRX N-terminal, and BRX domains of the CmRCC1 protein (Supplementary Figure S1A).^1^ Moreover, a database (The Arabidopsis Information Resource) search indicated 24 RCC1 family proteins in *A. thaliana*, among which 15 protein members have been named and functionally annotated. The phylogenetic tree built from the alignment of CmRCC1 with the previously identified *Arabidopsis* RCC1s revealed the evolutionary distances between the sequences (Figure 1A). Among these sequences, CmRCC1 showed high similarity to the sequences of AtRLD1 and AtRLD4.

The GFP-CmRCC1 fusion construct and GFP control in the pCAMBIA1305.4-N-GFP vector driven by *CaMV35S* promoter were transiently expressed in tobacco epidermal cells and visualized under a laser scanning confocal microscope to determine the subcellular localization of CmRCC1. The GFP fluorescence signal of GFP-CmRCC1 fusion protein was detected in the nucleus as confirmed by DAPI staining (Figure 1B).

### Temporal and Spatial Responses of *CmRCC1* Expression to Cold Stress

**Figure 2:**
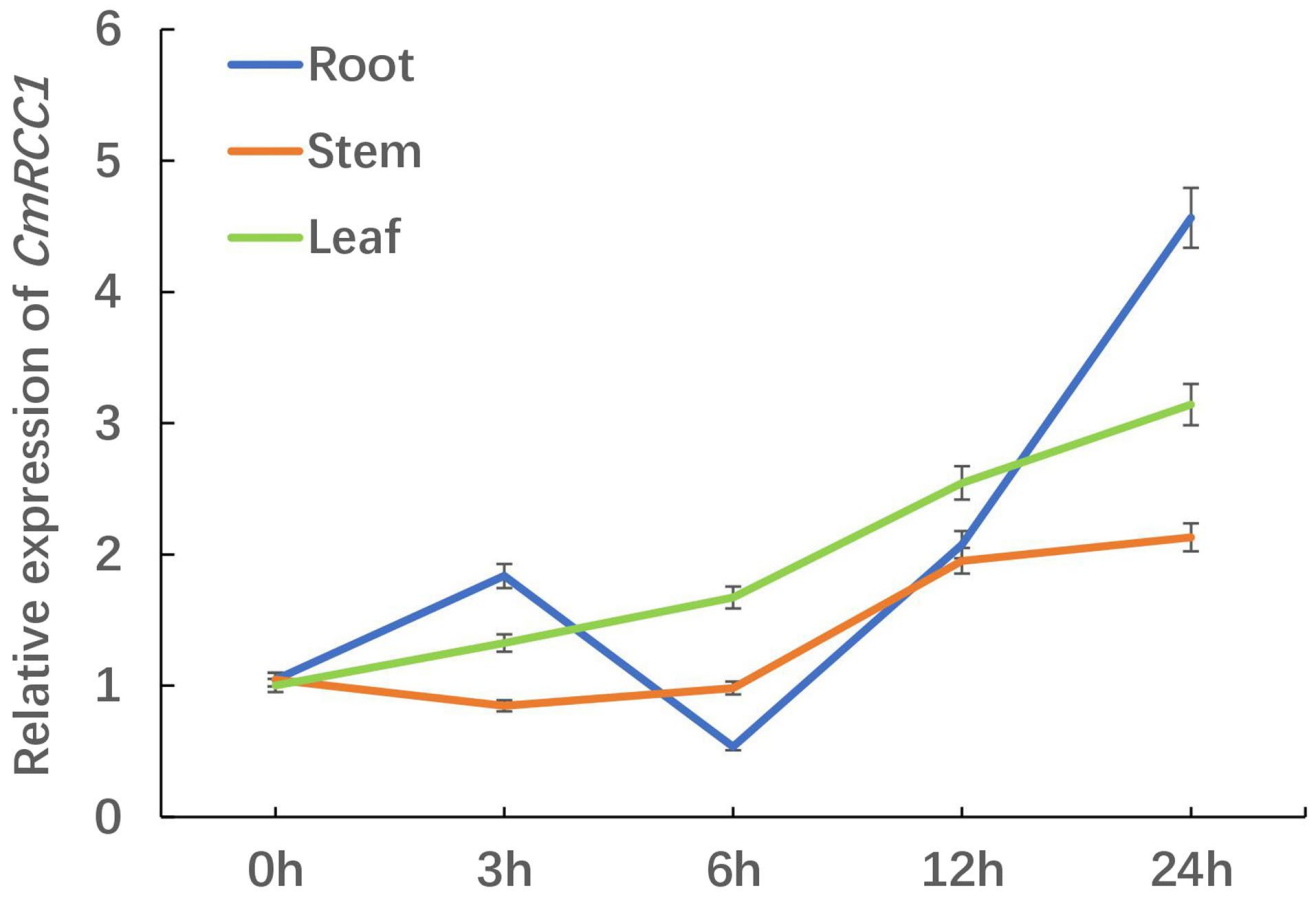
The time-course response in *CmRCC1* gene expression to chilling stress in pumpkin. Root, stem, and leaf samples were collected at the indicated times under chilling stress. The data are the means of four replicates with SEs.

We detected the changes in *CmRCC1* expression in the root, stem, and leaf at different time points after 24h cold treatment to evaluate the response characteristics of *CmRCC1* to cold stress in pumpkin. The transcription levels of *CmRCC1* in the stem and leaf increased slowly with the extension of cold stress treatment, and they reached 2.13 and 3.15 times of the control (0h) after 24h treatment, respectively. However, the expression level of *CmRCC1* in the pumpkin root peaked at 3h, and then reached 4.57 times at 24h of cold treatment (Figure 2). These results indicate that *CmRCC1* may be involved in the response of pumpkin root to early cold stress.

### Involvement of CmRCC1 in the Control of Root Architecture and the Regulation of *PIN* Gene Expression

**Figure 3:**
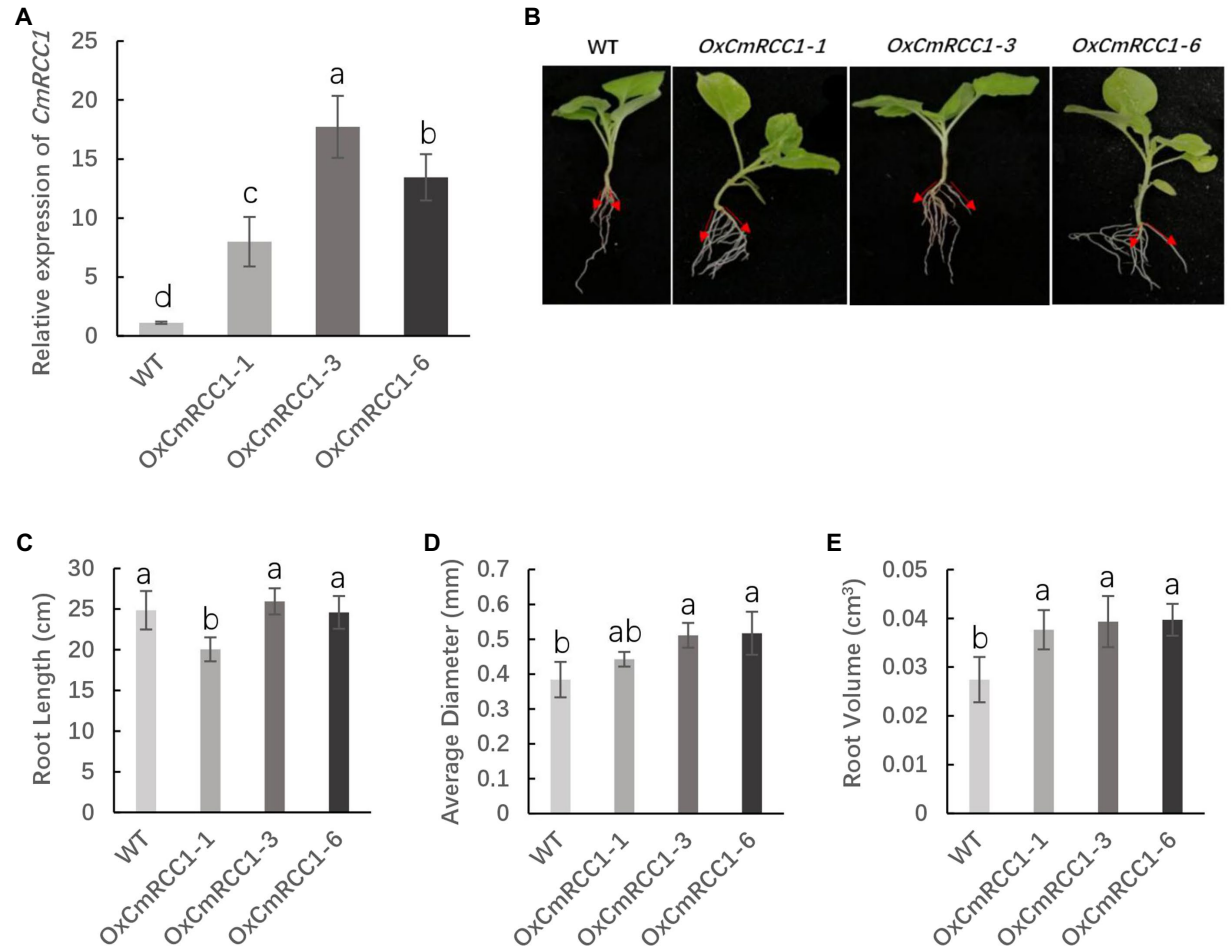
T_2_ generation phenotypes of three lines in overexpressed *CmRCC1* transgenic tobacco. **(A)** Relative expression level of *CmRCC1* in three different transgenic tobacco lines. **(B)** Gravitropic set-point angle (GSA) in lateral roots of WT and transgenic tobacco (*OxCmRCC1-1*/*−3/−6*). **(C)** Total root length in WT and transgenic lines. **(D)** Average root diameter in WT and transgenic lines. **(E)** Total root volume in WT and transgenic lines. WT, wild type. Samples were collected at the 4-week-old seedling stage. The data are the means of four replicates with SEs. Different letters indicate significant differences according to Tukey’s test (*p*<0.05).

*CmRCC1* was overexpressed in tobacco under the control of *CaMV35S* promoter to analyze the role of *CmRCC1* in root development. The insertion of the *CmRCC1* cassette in 28 independent kanamycin-resistant transformants was confirmed by RT-PCR (Supplementary Figure S2). Three transformed lines (*OxCmRCC1-1*/*−3/−6*) which showed that the *CmRCC1* gene segregated in the Mendelian segregation ratio of 3:1, were subsequently selected to obtain T_2_ homozygous lines (Supplementary Table S2). qRT-PCR analysis of the *CmRCC1* transcripts in three independent lines revealed variable levels of transgene expression (Figure 3A). Compared with the wild type, all the overexpressed transgenic lines showed increased gravitropic set-point angle (GSA) in lateral roots (Figure 3B). Moreover, *CmRCC1* overexpression increased the root diameter and volume of transgenic tobacco but not root length (Figures 3C–E).

**Figure 4:**
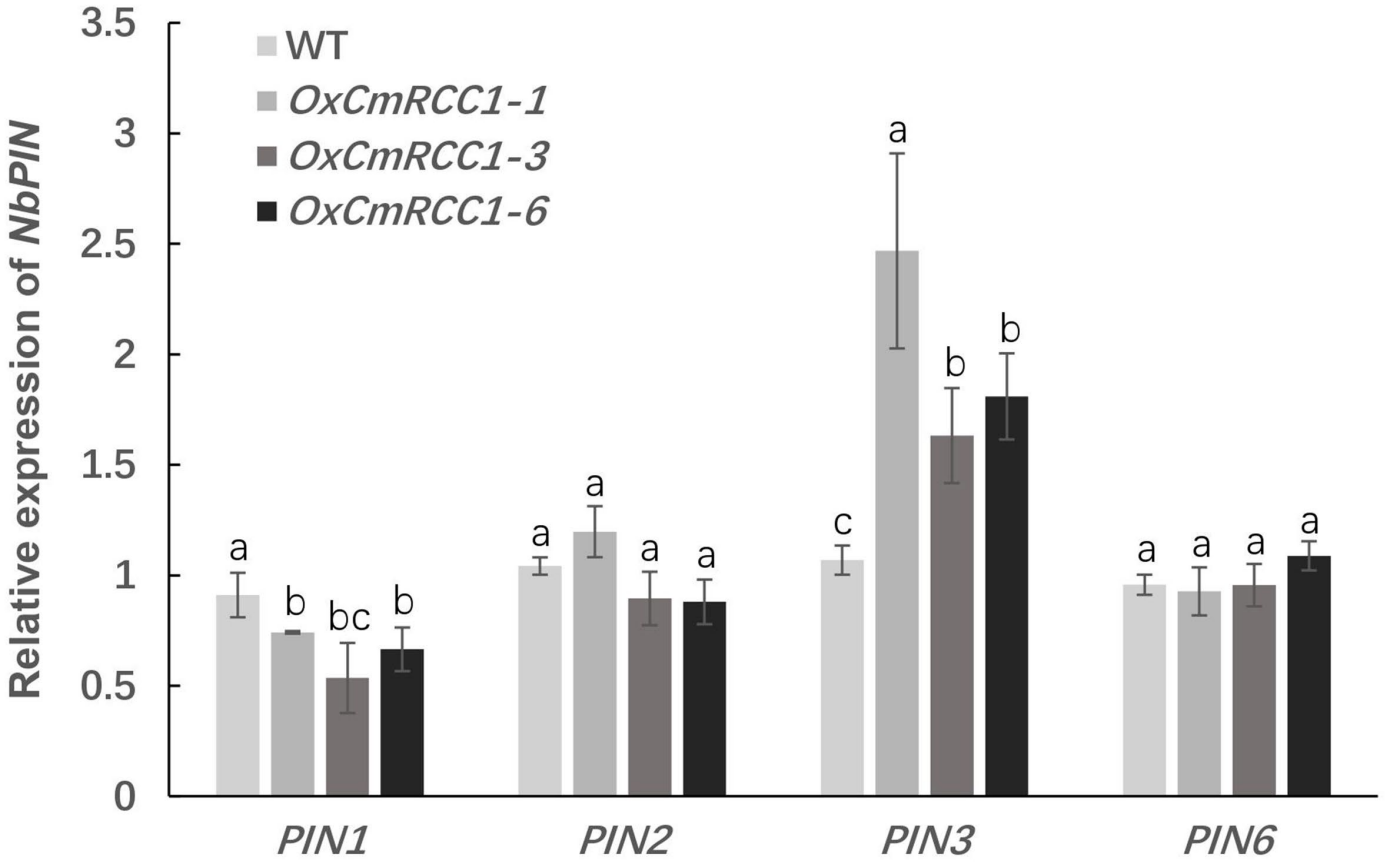
Expression analysis of the PIN family genes in transgenic tobacco. Root samples were collected at the 4-week-old seedling stage. Data represent means and SE of four replicates. Different letters indicate significant differences according to Tukey’s test (*p*<0.05).

In *Arabidopsis*, the characterized PIN proteins demonstrate specific expression patterns and are involved in polar auxin transport and root patterning (Paponov et al., 2005). Thus, we further measured the expression levels of four *PIN* genes in the roots of wild-type and *CmRCC1* transgenic plants. As shown in Figure 4, *PIN3* expression level remarkably increased in the *CmRCC1* overexpression lines than in the wild type. However, the expression of *PIN2* and *PIN6* showed no substantial differences between the transgenic lines and wild type. By contrast, the expression level of *PIN1* differentially decreased in the *CmRCC1* overexpression lines compared with the wild type.

### Interaction of CmRCC1 With CmLAZY1 Protein

**Figure 5:**
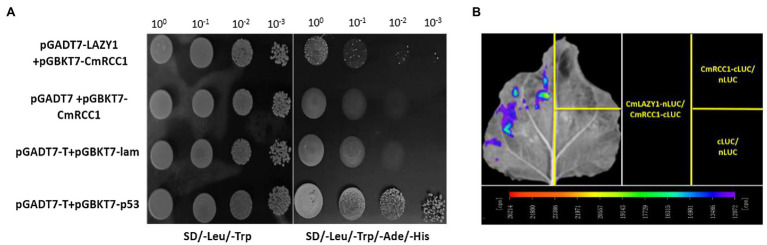
Interactions between CmRCC1 and CmLAZY1. **(A)** Interactions between CmRCC1 and CmLAZY1 in the yeast two-hybrid system. Recombinant plasmids containing either pGADT7-T and pGBKT7-p53 or pGADT7-T and pGBKT7-lam were introduced into yeast Y2H Gold cells and used as positive and negative controls, respectively. Yeast cells were cultured on SD/−Leu/−Trp and SD/−Leu/−Trp/−Ade/-His media. **(B)** Interactions between CmRCC1 and CmLAZY1 assayed with the luciferase complementation imaging assay. Tobacco leaves were divided into three parts and infiltrated with *Agrobacterium* strains harboring CmLAZY1-nLUC and CmRCC1-cLUC. The following two pairs of constructs were used as negative controls: CmRCC1-cLUC/nLUC and cLUC/nLUC. The images were captured with a charge-coupled device camera at 3days post-inoculation (dpi).

LAZY1 functions upstream of lateral auxin translocation in gravity signal transduction in the root and shoot of *Arabidopsis* and rice (Yoshihara and Iino, 2007; Taniguchi et al., 2017). We co-transformed pGADT7-CmLAZY1 and pGBKT7-CmRCC1 in yeast cells and found that the transformants grew on SD/−Leu/−Trp/−Ade/-His media, which was consistent with the results of the positive control yeast cells (Figure 5A). Furthermore, we performed luciferase complementation imaging assay to verify the interaction of CmRCC1 with CmLAZY1 *in vivo*. We were able to image LUC signals in tobacco leaves that co-infiltrated with *Agrobacterium* strains that expressed CmLAZY1-nLUC and CmRCC1-cLUC, but no signal was observed in the negative controls (CmRCC1-cLUC/nLUC and nLUC/cLUC, Figure 5B). Together, the results suggest that CmRCC1 interacts with CmLAZY1 protein.

### Increased Cold Tolerance in Transgenic Tobacco With *CmRCC1* Overexpression

**Figure 6:**
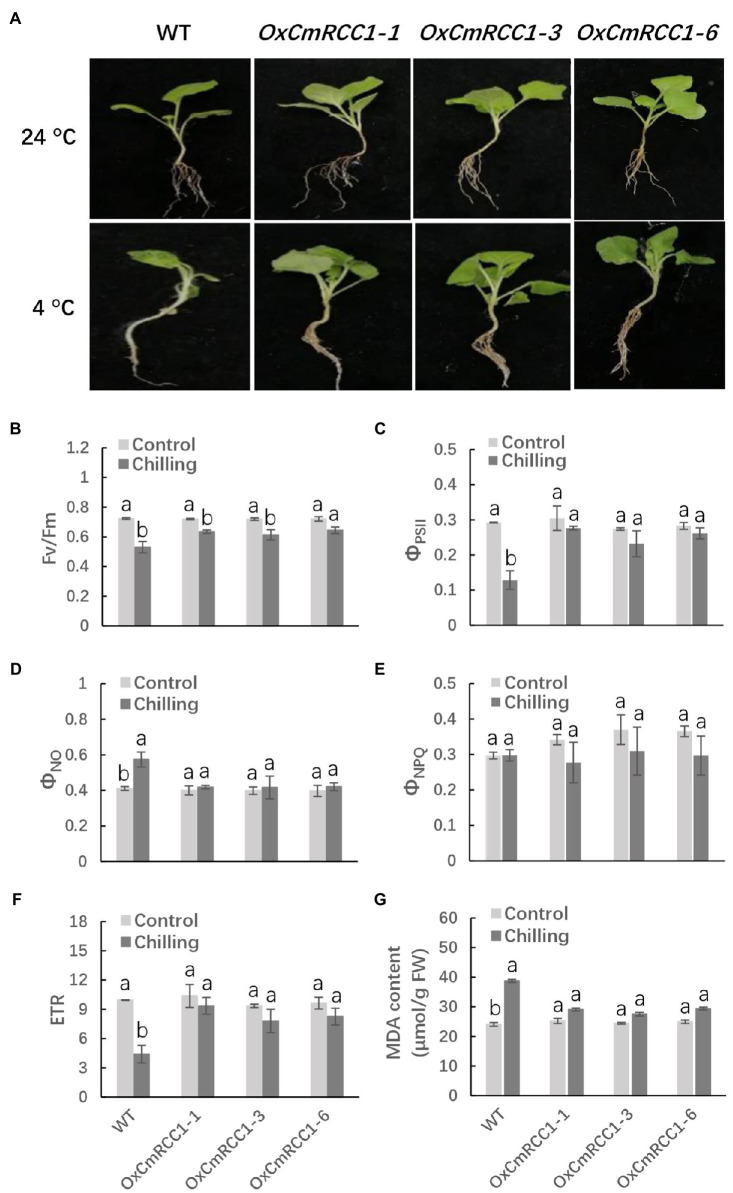
Chilling tolerance phenotypes in wild type (WT) and *CmRCC1* transgenic plants. **(A)** Phenotypes of 4-week-old WT and transgenic plants under normal (24°C) and chilling stress (4°C) conditions. The picture of representative plants was taken after 12h of 4°C treatment. **(B)** The maximum quantum yield of PS II (*F*v/*F*m). **(C)** The effective quantum yield of PS II (*Φ*_PSII_). **(D)** The quantum yield of nonregulated energy dissipation in PS II (*Φ*_NO_). **(E)** The quantum yield of regulated energy dissipation in PS II (*Φ*_NPQ_). **(F)** The electron transfer rate (ETR) at saturated light. **(G)** Malondialdehyde (MDA) content. Leaf samples were collected after 12h of 4°C treatment for chlorophyll fluorescence analysis. The data are the means of four replicates with SEs. Different letters indicate significant differences between the treatments according to Tukey’s test (*p*<0.05).

The seedlings of T_2_ transgenic lines and wild type were exposed to chilling stress at 4°C for 12h to examine the possible role of *CmRCC1* overexpression in the cold tolerance of tobacco. We observed that the leaves in the wild type completely shrank, and the plants were lodging after chilling stress treatment, whereas the transgenic tobacco plants still stood upright with flat leaves and light wilting (Figure 6A). We then measured the chlorophyll fluorescence of PS II in the third leaves of chilling-stressed and non-stressed plants in the wild-type and transgenic lines. The *F*v/*F*m and *Φ*_PSII_ decreased by 28.6 and 56.7%, respectively, in the wild type after chilling stress in comparison with the control. However, *F*v/*F*m and *Φ*_PSII_ decreased by 11.1–14.7 and 6.7–15.3%, respectively, in the *CmRCC1*-overexpressed lines in response to chilling stress (Figures 6B,C). A high *Φ*_NO_ value indicates that photochemical energy conversion and protective regulatory mechanisms are inefficient. Therefore, it indicates that the plant is already damaged or will be photodamaged upon further irradiation. Here, we found *Φ*_NO_ increased by 36.6% after chilling stress in wild-type plants, whereas *CmRCC1* overexpression compromised the increase in *Φ*_NO_ in chilling-stressed plants (Figure 6D). By contrast, *Φ*_NPQ_ showed no substantial differences between chilling-stressed and non-stressed plants in wild-type and *CmRCC1* transgenic lines, which indicates that the photoprotection ability was not affected under chilling stress (Figure 6E). We also analyzed the ETR versus incident photosynthetic photon flux density. Light-saturated ETR decreased by 55.0% in chilling-stressed wild-type plants. Again, the decrease in ETR was compromised in *CmRCC1*-overexpressed lines (Figure 6F). Moreover, increased MDA content (62.5%) was observed after 12h of chilling stress in wild-type plants compared with the control. However, no remarkable differences in MDA content were observed between the control and chilling-stressed transgenic lines (Figure 6G). Thus, we conclude that *CmRCC1* overexpression increases the cold tolerance of transgenic tobacco.

## Discussion

Vegetable crops, particularly those from the Cucurbitacaeae and Solanaceae families, are extensively grafted for increased yield and enhanced stress tolerance (Gaion et al., 2018). Facility cultivation producer would benefit from grafting to rootstocks that confer abiotic stress (i.e., cold) tolerance, which offer protection from soil-borne pathogens and maximize output by increasing yield (Williams et al., 2021). The characterization and identification of resistance genes can amplify the contribution of a breeding program to improve rootstock resistance.

RCC1 is a eukaryotic protein with seven repeated domains that fold into a seven-bladed propeller structure (Renault et al., 1998). RCC1-like domains (RLDs) have been identified in a variety of proteins that mediate diverse biological processes (Hadjebi et al., 2008). Two *Arabidopsis* RCC1 family proteins, UVR8 and TCF1, mediate UV-B response and tolerance to low temperature, respectively (Brown et al., 2005; Ji et al., 2015). Here, we show that the CmRCC1 protein plays a crucial role in the cold tolerance of transgenic tobacco. CmRCC1 shares conserved RCC1 repeat domains with the characterized *Arabidopsis* RCC1 family proteins, although the proteins differ concretely in sequence (Supplementary Figure S1). Similar to TCF1, CmRCC1 is localized in the nucleus, and the gene expression of *CmRCC1* is responsive to cold stress (Figures 1B, 2), which suggest a similar role of CmRCC1 during cold tolerance.

Photosynthesis is particularly sensitive to chilling during plant growth and development (Ruelland et al., 2009). Photosynthetic light harvesting is regulated by nonphotochemical quenching (NPQ), which allows the dissipation of harmful excess energy as heat through its energy-dependent NPQ (qE) component to avoid photodamage under chilling stress (Li et al., 2009; Niyogi and Truong, 2013; Ruban, 2016; Lu et al., 2020). In the green alga *Chlamydomonas reinhardtii*, UVR8 induces the accumulation of specific members of the light-harvesting complex (LHC) superfamily, particularly LHC Stress-Related 1 and Photosystem II Subunit S, which contribute to qE and reduce photodamage to the photosynthesis machinery under UV-B (Allorent et al., 2016). Our study showed that photoinhibition and photodamage around PS II were compromised in the *CmRCC1*-overexpressed lines under chilling stress (Figures 6B–D,F), which reveals a promising role of CmRCC1-mediated photoprotective regulation of photosynthetic activity in the chloroplast during chilling stress. Interestingly, although an excessive photon flux density occurs in the cold and night (Wise, 1995), the present results showed that the wild-type and transgenic plants retained some physiological means to protect themselves against excess light intensity during chilling in the light (Figure 6E).

A recent study indicated that RLD proteins, identified as LZY interactors, are essential regulators of polar auxin transport and root branch angle control (Furutani et al., 2020). Phylogenetic analysis revealed closer evolutionary distances between CmRCC1 and RLD family proteins (Figure 1A). Our results indicated that *CmRCC1* overexpression increased the GSA in lateral roots (Figure 3B), and the *in vitro* and *in vivo* interactions of CmRCC1 with CmLAZY1 protein suggest a possible role of CmRCC1 in the GSA control of lateral roots (Figure 5). Auxin is an important internal positive regulator during lateral root development, and genes of the PIN family have an important role in adaptation to stress responses through modulation in root system (Shibasaki et al., 2009; Wang et al., 2015; Zwiewka et al., 2019). *CmRCC1* overexpression induced decreased *PIN1* expression and increased *PIN3* expression in transgenic tobacco (Figure 4), which imply the differential roles of PIN family genes in the gravitropism regulation of lateral roots (Rosquete et al., 2013). In addition to GSA, the length, diameter, and volume of root components determine root system architecture (RSA). The exposure of monocot and dicot plant roots to temperatures below or above their optimum temperature decreases (i) primary root length, (ii) lateral root density (numbers of lateral roots per unit primary root length), and (iii) the branching angles between primary and lateral roots, whereas the average lateral root length is unaffected (Mcmichael and Quisenberry, 1993; Seiler, 1998; Nagel et al., 2009). In the present study, transgenic tobacco lines overexpressing *CmRCC1* exhibited increased root diameter and volume (Figures 3D,E), which help improve the soil volume that roots may access for the uptake of water and nutrients and further guarantee plant cold tolerance. Several NAC-type transcription factors from *Glycine max* were recently reported to increase lateral root formation by regulating the expression of auxin signaling-related genes, and improved cold tolerance was induced in transgenic plants with *GmNAC20* overexpression (Yang et al., 2019; Yarra and Wei, 2021).

We conclude that *CmRCC1* overexpression could enhance cold tolerance by improving RSA and maintaining photosynthetic activity under cold stress. Functional evidence on the role of root plasticity will support breeders in their efforts to include root properties in their future selection pipeline for cold stress tolerance to improve crop yield and quality.

## Data Availability Statement

The original contributions presented in the study are included in the article/Supplementary Material; further inquiries can be directed to the corresponding author.

## Author Contributions

FC and MW conceived and designed the research. MW, SZ, JL, and AX performed the experiments and analyzed the data. YH and ZB supervised the study. FC wrote the manuscript. All authors contributed to the article and approved the submitted version.

## Funding

This work was supported by the National Key Research and Development Program of China (2019YFD1000300), the Hubei Provincial Natural Science Foundation of China (2019CFB485), and the China Agriculture Research System of MOF and MARA (CARS-25).

## Conflict of Interest

The authors declare that the research was conducted in the absence of any commercial or financial relationships that could be construed as a potential conflict of interest.

## Publisher’s Note

All claims expressed in this article are solely those of the authors and do not necessarily represent those of their affiliated organizations, or those of the publisher, the editors and the reviewers. Any product that may be evaluated in this article, or claim that may be made by its manufacturer, is not guaranteed or endorsed by the publisher.

## Footnotes

^1^ http://pfam.xfam.org/search/sequence
