# Supplementary material for: CmRCC1 Gene From Pumpkin Confers Cold Tolerance in Tobacco by Modulating Root Architecture and Photosynthetic Activity
Source: Front Plant Sci. 2021 Dec 3;12:765302. doi: 10.3389/fpls.2021.765302 (PMC8678530; doi:10.3389/fpls.2021.765302)

Supplementary Material

CmRCC1 Gene from Pumpkin Confers Cold Tolerance in Tobacco by Modulating Root Architecture and Photosynthetic Activity

Running title: Molecular regulation of cold tolerance

Mengmeng Wang, Shu Zhou, Junyang Lu, Anqi Xu, Yuan Huang, Zhilong Bie, Fei Cheng*

Key Laboratory of Horticultural Plant Biology, Ministry of Education/College of Horticulture and Forestry Sciences, Huazhong Agricultural University, Wuhan, China

***Correspondence:** Fei Cheng, E-mail: feicheng@mail.hzau.edu.cn.

Table S1. List of primer sequences.

Gene name	Forward primer 5'-3'	Reverse primer 5'-3'
	For subcellular localization	
CmRCC1-N-GFP	Acggcatggacgagctgtacagatct<br>GCAAATGCCGACCGTGATGTC	Gccgggcggccgcttta<br>CTCACGGTTCTCTGACCACC
	For transgenic plants	
CmRCC1-pHellgate8	Catttggagaggacacgctcgag<br>TGCAAATGCCGACCGTG	Tctcattaaagcaggactctaga<br>GCTGGGAAGCTATTGAAACTGC
	For yeast two-hybrid	
pGBKT7-CmRCC1	Aggccgaattcccggggatccgt<br>GCAAATGCCGACCGTGATGTC	Ccgctgcaggtcgacggatccc<br>CTCACGGTTCTCTGACCACC
pGADT7-CmLAZY1	Gtgggcatcgatacgggatccgt<br>ATGAAGTTATTAGGATGGATGCACA	Cagctcgagctcgatggatcc<br>CTAAAGCTCCAACACCAAGT
	For luciferase complementation imaging assay	
pCAMBIA-CmRCC1-cLUC	Tcccggggcggtacc<br>GCAAATGCCGACCGTGATGTC	Gctctgcaggtcgac<br>CTCACGGTTCTCTGACCACC
pCAMBIA-CmLAZY1-nLUC	Tcccggggcggtacc<br>ATGAAGTTATTAGGATGGATGCACA	Gctctgcaggtcgac<br>CTAAAGCTCCAACACCAAGT
	For qRT-PCR	
CmRCC1	GGTGGTCAGAGAACCGTGAG	CTGTGACGACAGTCCCGAAA
CmCAC	GGACAAACAGAACCAACCATGA	GGTTTCCTTTCCGTCACTGTAGA
NbPIN1	CCGATATACTGAGCACAGGGG	ACTGAGAAGTGAGAGCTTAGCA
NbPIN2	ATGCTAGTTGCCTTGCCCATT	TCTTTCTTTCATGAATCCAGTGGC
NbPIN3	AATAACGGTTTTTCTTCTCCCAC	CCACCAACGAACAGAACCATA
NbPIN6	TGTTTCGGCAGCAATCTCCA	TCAATAGCTCCTCCTTGCACT
NbACTIN	TGGTCGTACCACCGGTATTGTGTT	TCACTTGCCCATCAGGAAGCTCAT

Table S2. Segregation of kanamycin resistance in *CmRCC1* transgenic T_1 generation lines.

Line	R	S	Total	Segregation Ratio
OxCmRCC1-1	26	10	36	3:1
OxCmRCC1-3	23	8	31	3:1
OxCmRCC1-6	28	9	37	3:1

R, positive plants with kanamycin resistance; S, negative plants with kanamycin sensitivity.

Figure S1. CmRCC1 protein domain prediction and alignment with RLD family proteins in *Arabidopsis*. (A) Online Pfam software (http://pfam.xfam.org/search/sequence) was used to predict conserved domains of CmRCC1 protein. **(B)** Multiple sequence alignment of CmRCC1 with RLD family proteins in *Arabidopsis* using Geneious software. The sequences in the blue box indicate the RCC1 repeats domain.

A

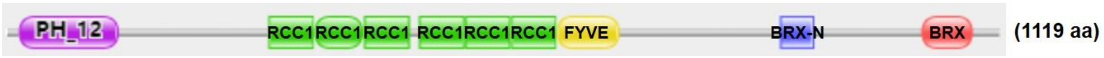

B

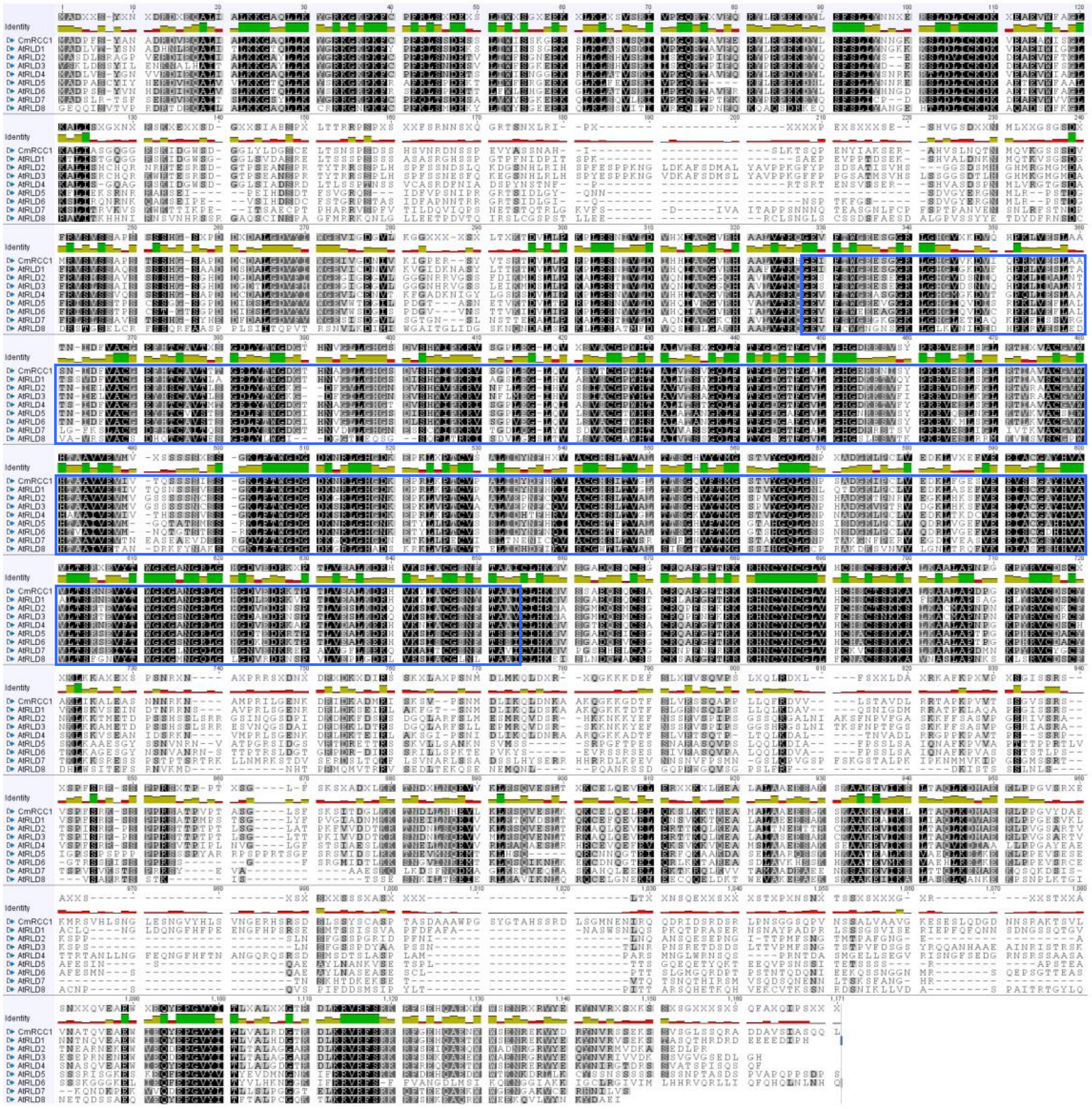

Figure S2. Identification of *CmRCC1* in transgenic tobacco lines. RT-PCR was performed to detect *CmRCC1* in transgenic T_0 generation lines with the primers from pHellgate8 vector and downstream of *CmRCC1* coding region, respectively.

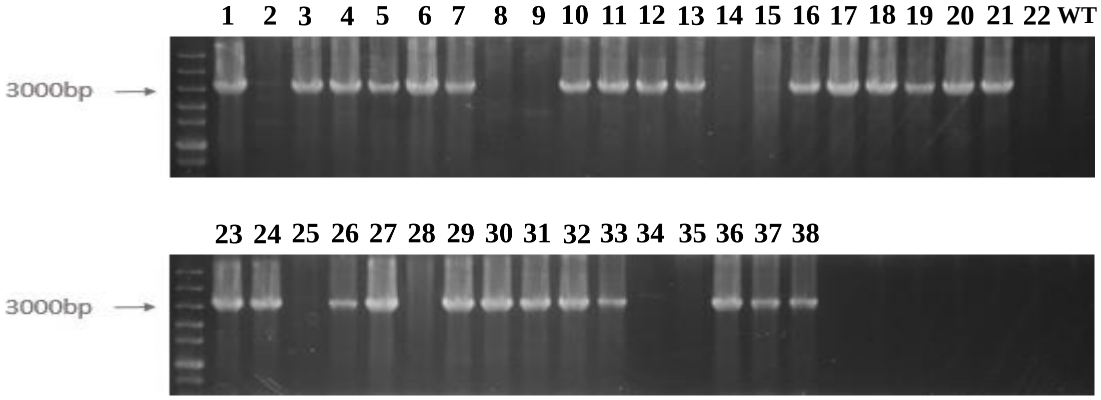

Supplement: Supplementary file 1 [file Data_Sheet_1.pdf]
